# Supplementary material for: The Spliceosome: A New Therapeutic Target in Chronic Myeloid Leukaemia
Source: Cancers (Basel). 2022 Sep 27;14(19):4695. doi: 10.3390/cancers14194695 (PMC9563771; doi:10.3390/cancers14194695)
Supplement: Supplementary file 1 [file cancers-14-04695-s001.zip › Supplemental materials.pdf]

Supplementary information

Supplementary Methods

SR1H4 protein staining

The phosphorylation status of SR proteins was assessed in K562 cells with the specific monoclonal antibody 1H4 (Santa Cruz Biotechnology, Dallas, United States) that recognizes different phosphorylated SR proteins. Briefly, $1x10^6$ cells were fixed using BD Fix and Lyse buffer, and then permeabilized with BD Phosflow Perm Buffer III (Becton Dickinson, Le Pont de Claix, France). Cells were washed twice with stain buffer and incubated at room temperature with the 1H4 antibody or an isotype matched control (Becton Dickinson) for 1h. After staining, cells were successively washed with stain buffer and PBS before analysis. Results were expressed as the ratio between the mean fluorescence intensity (MFI) of the labelled sample and the MFI of the isotype control.

rhAmp genotyping

Genomic DNA from primary CP-CML cells was analysed using custom rhAmp™ SNP assays (Integrated DNA Technology). Briefly, locus and allele specific primers were generated individually for RNU1_Batch and RNU1_Pseudo. Assays were run in technical duplicates in 5µL volume (DNA concentration: at least 5ng/µL), with control gBlocks for wildtype, mutant and heterozygous genotypes. Reporter mix used the Yakima Yellow (mutant) and FAM (wild type) dyes, and also the ROX dye for passive reference. Plates were read on the QuantStudio 6 (ThermoFisher) real-time PCR instrument, and genotypes called using the QuantStudio 6 and 7 Flex Real-Time PCR System Software. The primer sequences are available in **Supplemental Table 4**.

Next Generation Sequencing (NGS)

A capture-based commercial solution was used (Myeloid Solution™ SOPHiA GENETICS, Saint Sulpice, Switzerland). Sequencing was performed on an Illumina MiSeq sequencing platform, and results were analysed using the SOPHiA DDM® platform (see panel in **Supplemental Table 5)**.

Supplemental Figures and tables legends

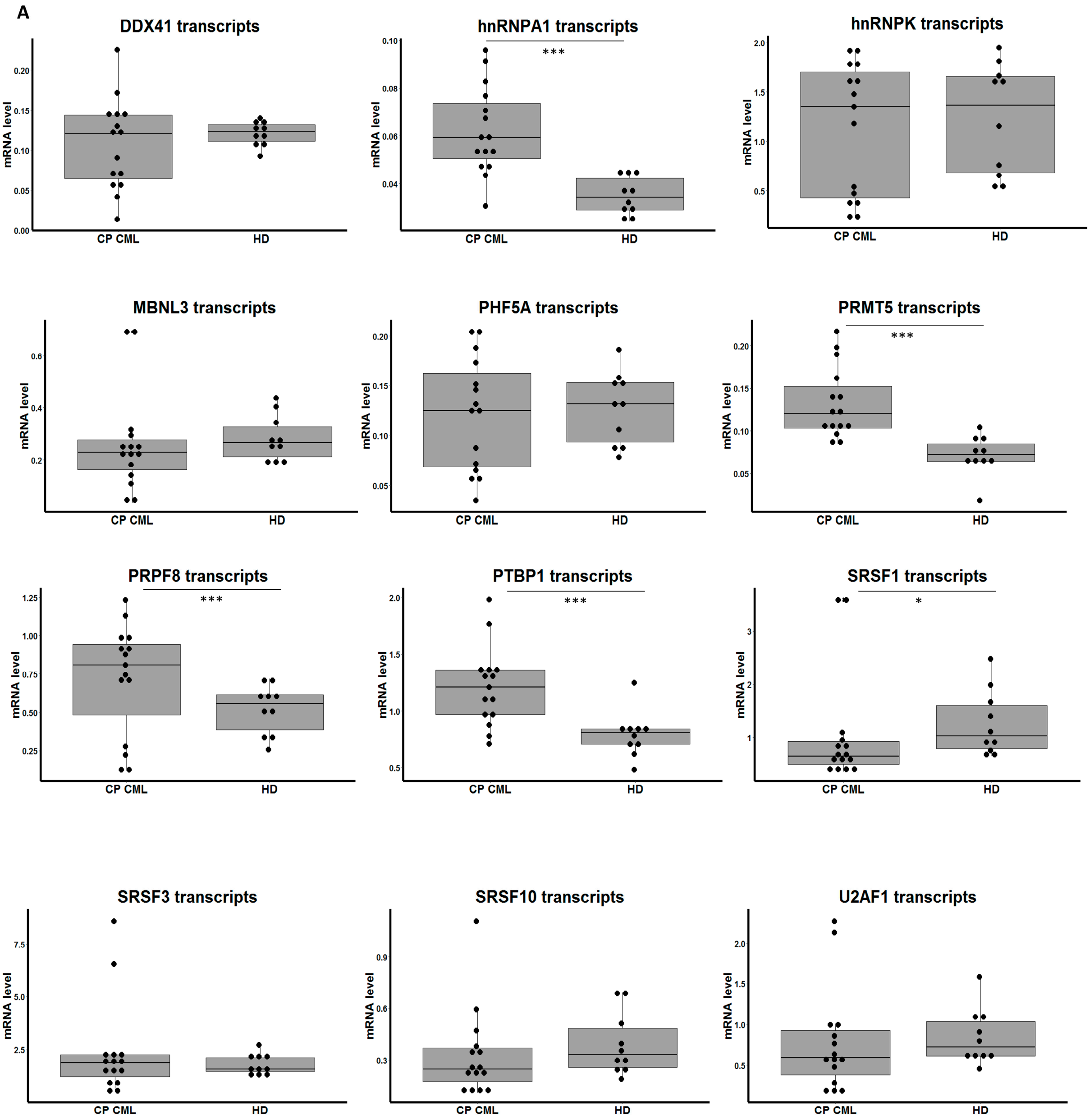

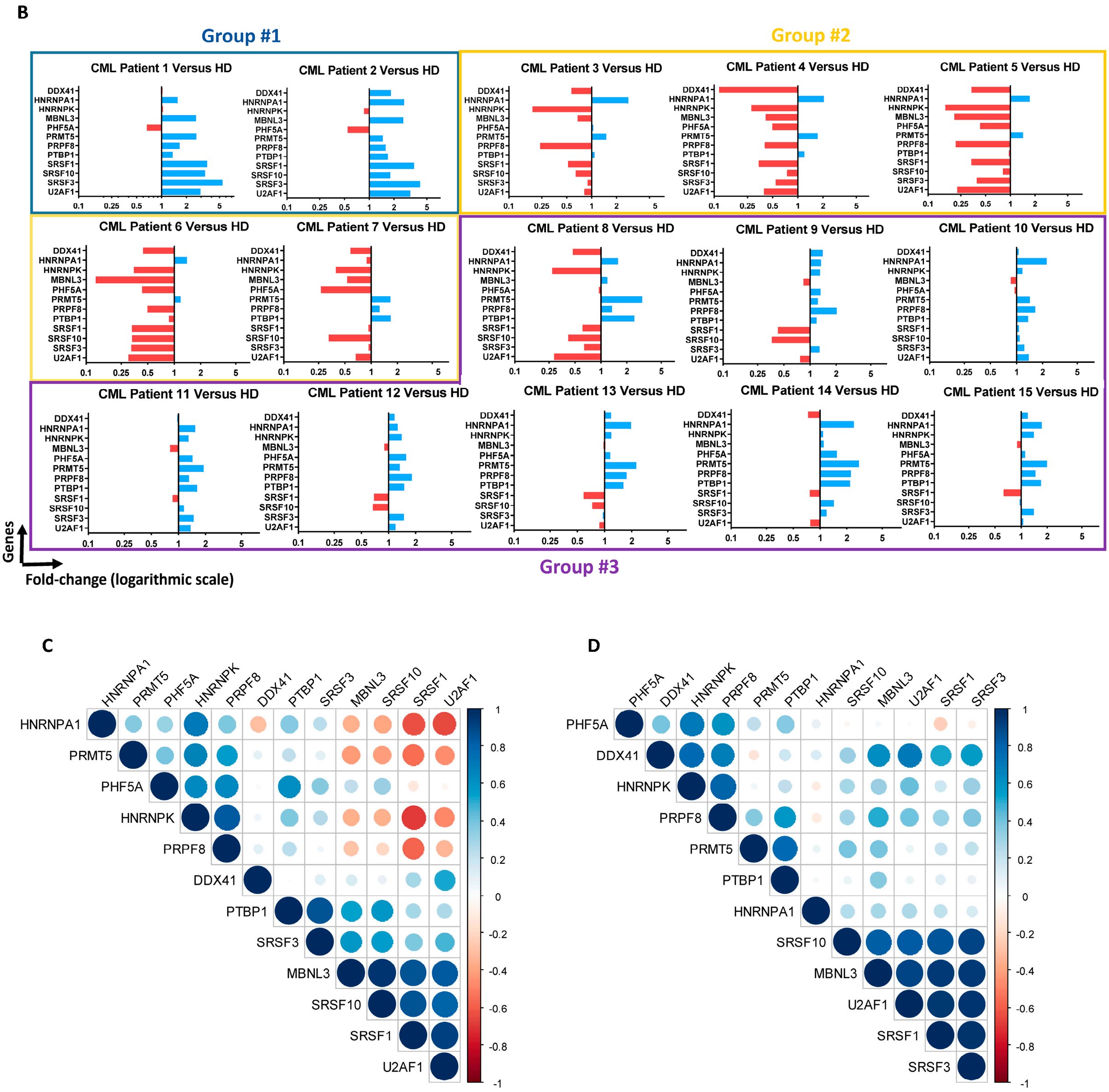

Supplemental Figure S1: mRNA transcript levels in CP-CML Versus HD CD34+CD15- cell samples

Gene expression was assessed using the Fluidigm BioMark HD System in $CD34^{+}CD15^{-}$ cells from patients with chronic phase chronic myeloid leukaemia (CP-CML) (n=15) and healthy donors (HD) (n=10). A. Expression of the 12 splicing genes in CP-CML and HD cell samples; B. Three patient groups defined on the basis of the expression profile of the 12 genes implicated in splicing; C. Correlation matrix of spliceosome gene expression in HD $CD34^{+}$CD15- cells; D. Correlation matrix of spliceosome gene expression in CP-CML $CD34^{+}$CD15- cells.

*p <0.05, **p < 0.01, ***p <0.001 (Kruskal-Wallis test).

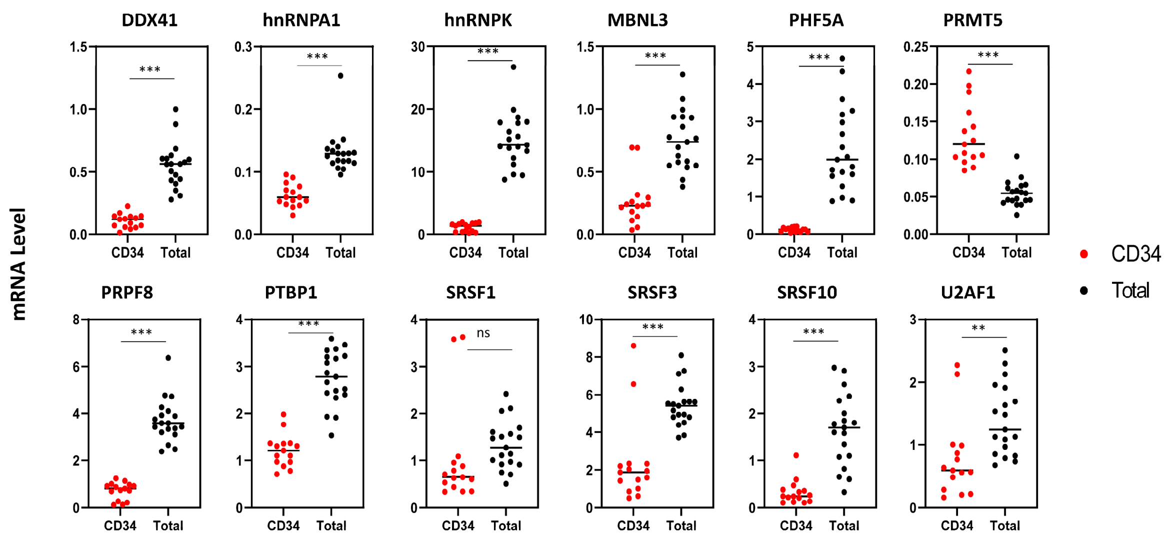

Supplemental Figure S2: Gene expression in CD34+CD15- cells and total blood cells from patients with CP-CML

Expression of the 12 genes implicated in splicing was assessed using the Fluidigm BioMark HD System in $CD34^{+}CD15^{-}$ cells (n=15) and total peripheral blood leukocytes (n=19) from patients with CP-CML (not the same patients; see Supplemental Table 1).

**: p <0.01, ***: p <0.001, ns: not significant (t-test).

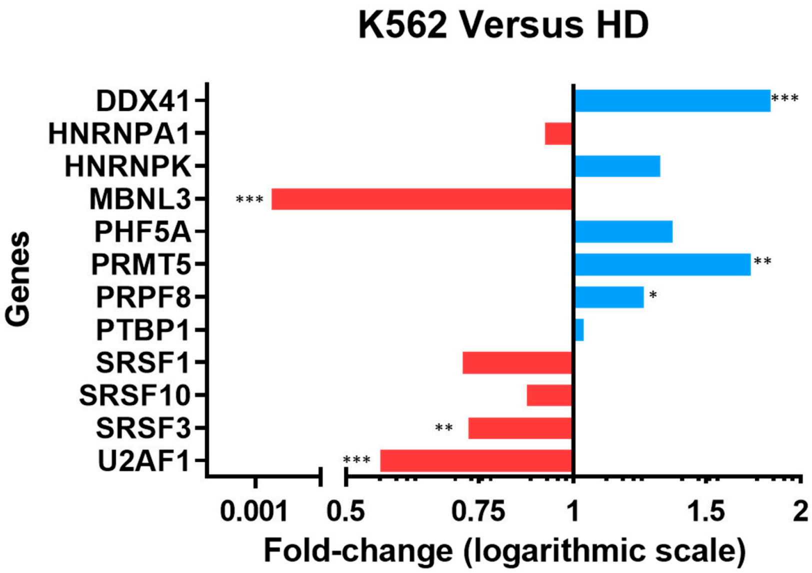

Supplemental Figure S3: Spliceosome gene expression in K562 cells (n=3) and $CD34^{+}CD15^{-}$ cells from healthy donors (HD) (n=10)

Red: downregulated genes and blue: upregulated genes *versus* HD cells.

*p <0.05; **p <0.01; ***p <0.001 (Kruskal-Wallis test).

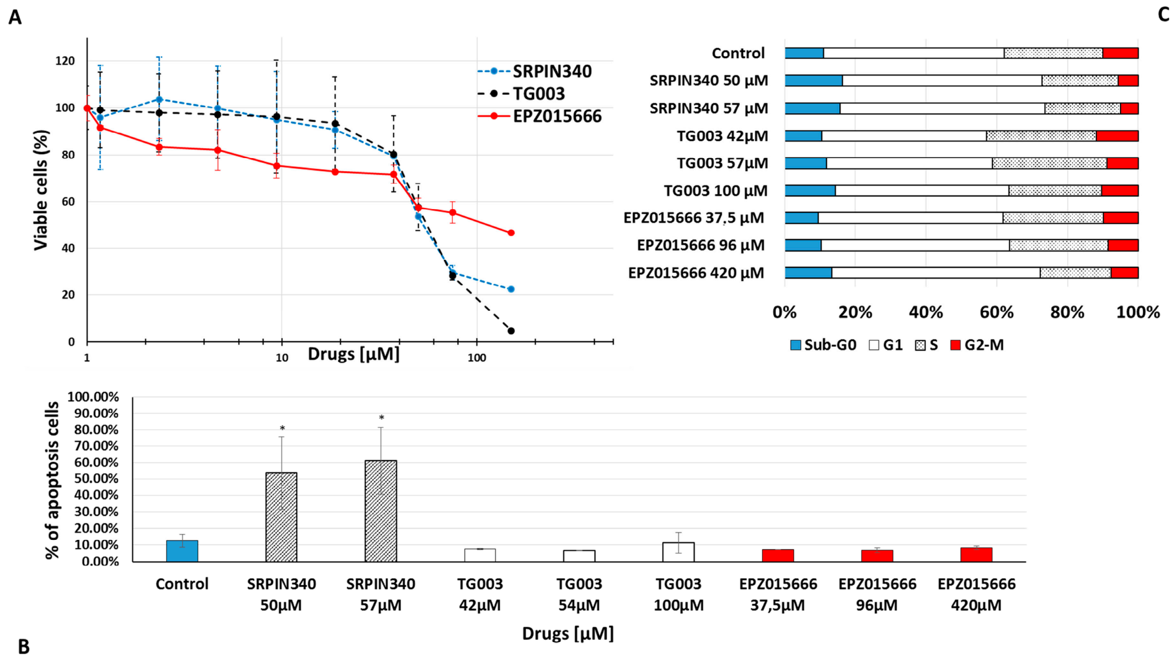

Supplemental Figure S4: Effect of spliceosome targeted drugs on KCL22 cell viability, death and cell cycle.

A. *KCL22 cells were incubated with increasing concentrations (0–300 µM) of SRPIN340, TG003, or EPZ015666 for 72h. Cell viability was determined using the resazurin assay. Viability of control cells (incubated with vehicle) was set at 100% and viability of treated cells was calculated relative to control cells. Values are the mean ± standard deviation of three independent experiments (n=3);* ***B.*** *To assess cell death, KCL22 cells were incubated with three concentration of each drug for 72h. Cells incubated with vehicle were used as controls. Then, cell death was evaluated by annexin V-FITC and 7-AAD staining. Values are the mean ± standard deviation of four independent experiments (n=3) ;* ***C.*** *To assess the effect of each drug on cell cycle, KCL22 cells were incubated with different concentrations of each drug for 24h. Cells incubated with vehicle were used as controls. The effect on cell cycle was evaluated by propidium iodide staining. Values are the mean of three independent experiments (n=2).*

**p <0.05 (t-test paired)*

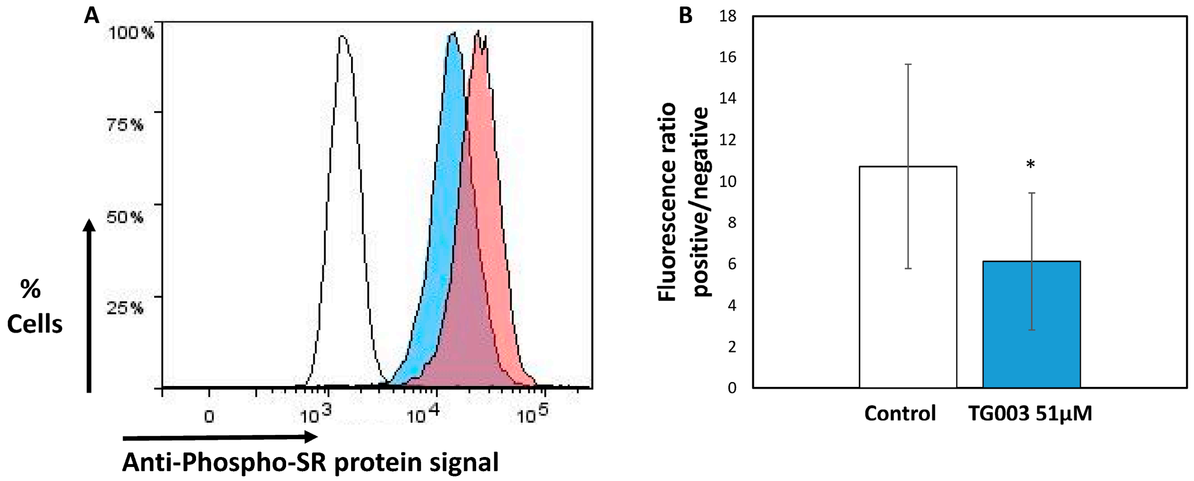

Supplemental Figure S5: TG003 inhibits SR protein phosphorylation in K562 cells

K562 cells were incubated with 51µM TG003 (IC50).

***A.** Representative experiment: negative signal (white), positive signal of control cells (red), and positive signal of cells incubated with TG003 (blue). **B.** Ratio of positive/negative signal in cells incubated or not (control) with TG003 (mean ± SD of 3 independent experiments).*

**p <0.05 (t test paired)*

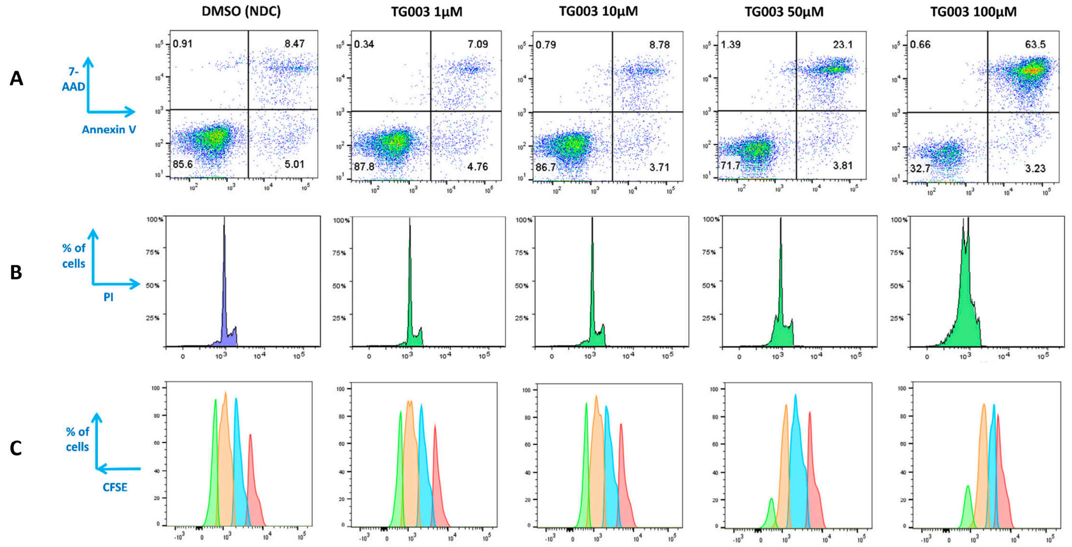

Supplemental Figure S6: Illustrative example of TG003 functional impact on primary CP-CML CD34⁺ cells

***A.** Representative flow cytometry dot plots of primary CP-CML CD34$^+$ cells showing the concentration-dependent apoptotic response to incubation with TG003 for 72h. **B.** Representative flow cytometry histograms of primary CP-CML CD34$^+$ cells showing the concentration-dependent cell cycle perturbation after incubation with TG003 for 72h. **C.** Representative flow cytometry histograms of primary CP-CML CD34$^+$ cells showing the concentration-dependent cell proliferation perturbation after incubation with TG003 for 72h.Red: position of viable CFSE-stained cells (undivided; division 0). Blue: cells that divided once; orange: cells that underwent two divisions; bright green: cells that divided three times.*

All cell samples were cultured for 72h in serum free medium with physiological growth factors with or without drugs at different concentrations. Patient n°27

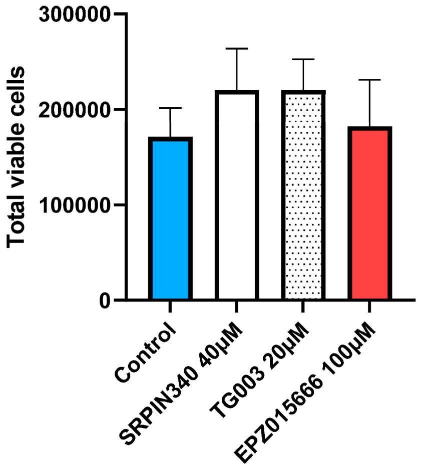

Supplemental Figure S7: SRPIN340, TG003 and EPZ015666 had no effect on cell viability in PBC from healthy controls

Total cells from healthy controls *were incubated with SRPIN340 (40µM), TG003 (20µM), and EPZ015666 (100µM) for 72h. Cell viability was determined using NucleoCounter NC-3000. Values are expressed as the mean ± standard deviation of three patients.*

Supplemental Table S1: Characteristics of patients with CP-CML

Supplemental Table S2: Primers sequences RT-qPCR

Supplemental Table S3: Primers sequences Fluidigm

Supplemental Table S4: rhAmp genotyping

Supplemental Table S5: NGS Panel
